# Development and Efficacy of Real-Time PCR in the Diagnosis of Vivax Malaria Using Field Samples in the Republic of Korea

**DOI:** 10.1371/journal.pone.0105871

**Published:** 2014-08-22

**Authors:** Jung-Yeon Kim, Youn-Kyoung Goo, So-Young Ji, Hyun-Il Shin, Eun-Taek Han, Yeonchul Hong, Dong-Il Chung, Shin-Hyung Cho, Won-Ja Lee

**Affiliations:** 1 Division of Malaria and Parasitic Diseases, National Institute of Health, Korea CDC, Osong Saeng-myeong 2 ro, Osong Health Technology Administration Complex 187, Osong, Republic of Korea; 2 Department of Parasitology and Tropical Medicine, Kyungpook National University School of Medicine, Daegu, Republic of Korea; 3 Department of Parasitology, Kangwon National University College of Medicine, Chuncheon, Republic of Korea; Ehime University, Japan

## Abstract

The development of sensitive, rapid, and accurate diagnostic methods for vivax malaria is essential for the effective control of malaria in the Republic of Korea, where vivax malaria patients usually show low parasitemia. In this study, a TaqMan-based real-time polymerase chain reaction (PCR) method was established and compared with other PCR-based assays, including nested PCR, loop-mediated isothermal amplification, and multiplex PCR, using samples from febrile patients with suspected vivax malaria. The established real-time PCR had a high sensitivity (99.6%) and specificity (100%). Therefore, this sensitive, specific, rapid, and quantitative real-time PCR method could be used for the routine diagnosis of vivax malaria in the laboratory of the Korea National Institute of Health.

## Introduction

Malaria caused by *Plasmodium* spp. occurs worldwide. According to the World Malaria Report published by the World Health Organization (WHO), 207 million malaria cases occurred among 3.3 billion at-risk people from 103 countries in 2012, resulting in an estimated 627,000 deaths [1]. In addition, malaria is re-emerging in areas where it had been previously eradicated, including Eastern Europe and Central Asia [2], [3]. *Plasmodium vivax* is the only indigenous malaria species in the Republic of Korea (ROK). Since its re-emergence in 1993, vivax malaria has become endemic in the ROK, with seasonal prevalence [4]. From 1994 to 2010, 29,692 cases of indigenous vivax malaria were reported in the ROK. The Korea Centers for Disease Control and Prevention (CDC) aims to eliminate malaria by 2015 by controlling the disease through early and accurate diagnosis as well as radical and rapid treatment [5], [6]. Therefore, the attainment of standardized and highly sensitive diagnostic methods is critical.

For the past 100 years, malaria has been diagnosed microscopically using Giemsa-stained thick and thin blood films [7]. This method is the gold standard for malaria diagnosis and is recommended by the WHO. However, several field studies have reported that microscopy results are inconsistent, depending on individuals performed. In addition, parasite detection poses a great challenge, especially when parasitemia is low [8]–[10]. On the other hand, several polymerase chain reaction (PCR)-based assays for malaria diagnosis have been developed, and are most often based on genus- or species-specific sequences of the small subunit (SSU) 18S rRNA genes of the parasite [11]–[14]. PCR-based assays have several advantages over microscopy: they are highly specific and sensitive [15]– and can detect less than one parasite per microliter of blood [19]. Furthermore, they are ideal for revealing the presence of mixed infections that are often missed by conventional methods [20]–[22].

The low parasitemia observed in vivax malaria often leads to misdiagnosis of infection in microscopic examination by clinicians. To date, nested PCR, loop-mediated isothermal amplification (LAMP), and real-time PCR methods have been developed to detect vivax malaria [14], [23], [24]. Compared with nested PCR, real-time PCR uses fluorescent labels that allow the continuous monitoring of amplicon (PCR product) formation throughout the reaction. Furthermore, automated real-time PCR is a rapid technique that does not require multiple and complex methods or skilled technologists, raising the possibility of being used for the sensitive and specific diagnosis of malaria. Other advantages of real-time PCR techniques include pathogen quantification, reduced risk of contamination, and availability of results within 2 hours.

However, monitoring data for the basic diagnosis of vivax malaria using PCR-based assays in the ROK are scarce. Therefore, the present study aimed to establish a TaqMan-based real-time PCR method and compare its limit of detection (LOD) with other PCR-based assays, i.e., nested PCR, LAMP, and multiplex PCR. In addition, the correlation between parasitemia and C_t_ values of the developed real-time PCR was evaluated. Finally, the sensitivity and specificity of the PCR-based assays were evaluated using samples from Korean patients with suspected vivax malaria.

## Materials and Methods

### Ethics statement

The study was approved by the research ethics committee of the Korea National Institute of Health. All participants signed a written informed consent form and agreed to provide a 5-mL blood sample.

### Blood samples and DNA extraction

A total of 319 venous blood samples from patients with symptoms of malaria were collected throughout the ROK between January and November 2012. The admission and clinical management of the patients were undertaken independently of this study. Among 319 samples, 192 samples transported within 6 hours (in fresh condition) or blood smears were assigned for microscopic examination. All 319 samples were tested using 4 PCR-based assays: nested PCR, real-time PCR, LAMP, and multiplex PCR. Aliquots (200 µL) of 319 venous blood samples in EDTA-coated tubes were stored at −20°C, and subsequently used for genomic DNA extraction using a QIAamp DNA Blood Mini Kit (Qiagen; USA) according to the manufacturer's instructions. After extraction, each genomic DNA sample was eluted in 50 µL of distilled water.

### Nested PCR

Nested PCR was performed with the extracted DNA using specific primers for the *Plasmodium* genus (rPLU 5 and rPLU 6) and *P. vivax* (rVIV 1 and rVIV 2), as previously described (Table 1) [14]. In brief, 5 µL of the extracted DNA was used as a template in the primary PCR with rPLU 5 and rPLU 6, and then 1 µL of the primary PCR product was used in the second amplification with rVIV 1 and rVIV 2. The final PCR products were resolved by electrophoresis on a 2% agarose gel, stained with SafePinky DNA Gel Staining Solution (GenDEPOT; USA), and visualized with ultraviolet illumination.

**Table 1:** Primers for nested PCR, TaqMan-based real-time PCR, and LAMP of the SSU 18S rRNA gene in *Plasmodium vivax*.

Type of PCR	Species	Primer or probe	Sequence (5′–3′)	Size of PCR product (bp)	Reference
Nested, first reaction	*Plasmodium* sp.	rPLU5	CTTGTTGTTGCCTTAAACTTC	1200	[9]
		rPLU6	TTAAAATTGTTGCAGTTAAAACG		
Nested, second reaction	*P. vivax*	rVIV1	CGCTTCTAGCTTAATCCACATAACTGATAC	120	
		rVIV2	ACTTCCAAGCCGAAGCAAAGAAAGTCCTTA		
Real-time	*P. vivax*	Plasmo-F	AAG TTA AGG GAG TGA AGA CGA TCA GA	170	
		Plasmo-R	AGA ACC CAA AGA CTT TGA TTT CTC ATA A		
		VIV probe (VIC)	VICTAA GAA TTT TCT CTT CGG AGT TTMGBNFQ		
LAMP	*P. vivax*	F3	GGAATGATGGGAATTTAAAACCT		[20]
		B3c	ACGAAGTATCAGTTATGTGGAT		
		FIP (F1c-F2)	CTATTGGAGCTGGAATTACCGCTCCCAAAACTCAATTGGAGG		
		BIP (B1-B2c)	AATTGTTGCAGTTAAAACGCTCGTAAGCTAGAAGCGTTGCT		
		LPF	GCTGCTGGCACCAGACTT		
		LPB	AGTTGAATTTCAAAGAATCG		

LAMP: loop-mediated isothermal amplification; PCR: polymerase chain reaction; SSU, small subunit.

### Real-time PCR

*P. vivax*-specific real-time PCR was performed on the 319 blood samples using the StepOne System (Applied Biosystems; USA). Primers (forward and reverse) and TaqMan fluorescence-labeled probes were designed using Primer Express software (Applied Biosystems; USA) to amplify the *P. vivax* SSU 18S rRNA gene. These primers (Plasmo-F and Plasmo-R) and probes (VIV probe) are listed in Table 1. The PCR assay was performed in a final volume of 20 µL containing 5 µL of the extracted DNA, 10 µL of TaqMan Universal Master Mix (Applied Biosystems; USA), 100 nM each of Plasmo-F and Plasmo-R primers, and 100 nM of VIV probe. All the real-time PCR assays, in parallel with nested PCR, were performed in duplicate. The PCR conditions consisted of an initial denaturation step at 95°C for 10 min, followed by amplification for 40 cycles of 10 s at 95°C, 5 s at 50°C, and 20 s at 72°C, with fluorescence acquisition at the end of each extension step. Amplification was immediately followed by a step for melting curve analysis consisting of 2 min at 95°C, 2 min at 68°C, and a stepwise temperature increase of 0.2°C/s to 90°C, with fluorescence acquisition at each temperature transition. The fluorescence data were analyzed using a cutoff of 35 cycles to identify *Plasmodium*-positive samples. Using results of the nested PCR as the gold standard, the sensitivity, specificity, and agreement values of the real-time PCR assay for each detected *P. vivax* were summarized.

### LAMP assay

LAMP assays were performed on the 319 blood samples using a protocol reported previously [25]. The primers used are listed in Table 1. Briefly, reaction mixtures were prepared with Loopamp DNA amplification kits (Eiken Chemical Co., Ltd.; Japan) as follows: 2.4 µM of both FIP and BIP primers, 0.2 µM of both F3 and B3c primers, 0.8 µM of LPF and LPB, 12.5 µL of 2× reaction mixture (40 mM Tris-HCl [pH 8.8], 20 mM KCl, 16 mM MgSO_4_, 20 mM (NH_4_)_2_SO_4_, 0.2% Tween 20, 1.6 M betaine, and 2.8 mM of each deoxyribonucleoside triphosphate), Bst DNA polymerase, and 5 µL of DNA sample. LAMP was performed at 60°C in a water bath for 60 min, and visibly turbid samples were considered positive, in a single blind manner. LAMP reaction mixes were stained with SYBR green I dye (Eugene, USA), and positive samples were identified with the naked eye under an ultraviolet transilluminator or using a handheld black light.

### Multiplex PCR

Multiplex PCR was performed using a commercial kit (DiaPlexC Malaria Detection kit, SolGent, ROK) according to the manufacturer's instructions. The commercial kit can detect four *Plasmodium* species—*P. vivax*, *P. falciparum*, *P. ovale*, and *P. malariae*—with a single reaction. In brief, 25-µL reaction mixtures were prepared by adding the following reagents: 12.5 µL of 2× multiplex PCR premix, 2 µL of primer mixture, 5 µL of the extracted DNA, and 5.5 µL of distilled water. The PCR cycle conditions were as follows: uracil DNA glycosylase activation at 50°C for 3 min; denaturation at 95°C for 15 min; amplification for 40 cycles at 95°C for 30 s, 62°C for 30 s, and 72°C for 40 s; and extension at 72°C for 5 min. The final PCR products were resolved by electrophoresis on a 1% agarose gel, stained with SafePinky DNA Gel Staining Solution (GenDEPOT; USA), and visualized with ultraviolet illumination.

### Microscopic examination

Microscopic examination for the LOD calculation was performed on blood samples collected from vivax malaria patients whose infection had been previously confirmed by nested PCR and microscopic examination. In addition, 192 out of 319 samples were tested by microscopic examination to evaluate the correlation between parasitemia and C_t_ values of the real-time PCR. Thick blood smears were prepared, dried, stained with 10% Giemsa, and examined microscopically. Parasite density was assessed by counting 200 leucocytes and converting the result to parasites per microliter by assuming a standard count of 8000 leucocytes/µL. Two technicians counted the parasites under blinded study conditions.

### LOD of three PCR methods

Two-fold serial dilutions (from 2^0^ to 2^13^ dilutions) were prepared for the vivax sample from a patient with known parasitemia (800 parasites/µL) diagnosed with thick smears. Nested PCR, real-time PCR, LAMP, multiplex PCR, and microscopic examination were conducted using this sample as described above. All PCR-based assays were performed in duplicate.

### Statistical analysis

The clinical sensitivity, specificity, and agreement of the real-time PCR, LAMP, and multiplex PCR assay for detecting *P. vivax* were calculated using nested PCR results as the reference standard. Sensitivity was calculated as follows: [number of true positives/(number of true positives + false negatives)] ×100. Specificity was calculated as follows: [number of true negatives/(number of true negatives + false positives)] ×100. Agreement was calculated using DAG_Stat and VassarStats (http://vassarstats.net/). To determine the statistical significance of the results, the confidence interval and margin of error for the sample size were determined.

## Results and Discussion

Vivax malaria in the ROK is characterized by low parasitemia and a long latency period after primary infection in humans. Owing to the low parasitemia, microscopic examination often results in misdiagnosis and discrepant results with other diagnostic methods even though it is still considered as the gold standard. As indicated in previous studies, PCR-based assays are more specific and sensitive than microscopic examination for detecting and identifying malaria parasites [26], [27]. Among the PCR-based assays, real-time PCR is as sensitive as conventional PCR to detect malaria parasites and enables parasite quantification [28]. To date, the efficacy of real-time PCR in the detection of vivax malaria has not been compared with that of other PCR-based assays (nested PCR, LAMP, and multiplex PCR) in field samples from the ROK. Therefore, we established a real-time PCR method to amplify the SSU 18S rRNA gene of *P. vivax* for routine diagnosis of vivax malaria in this laboratory and compared its diagnostic efficacy with those of other diagnostic tools—nested PCR, LAMP, and multiplex PCR—using field samples obtained from patients with suspected vivax malaria.

**Figure 1:**
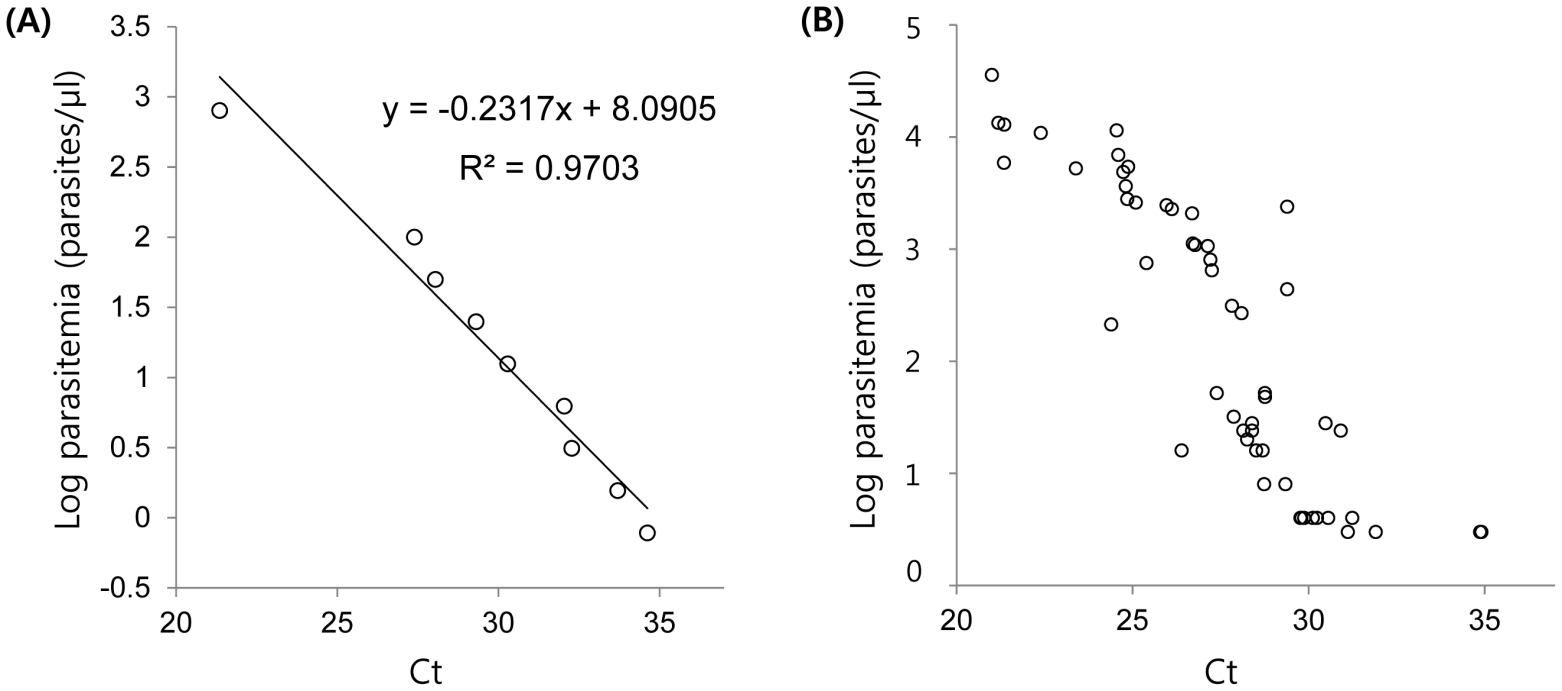
Sensitivity of the real-time polymerase chain reaction assay for detecting *Plasmodium vivax* in a blood sample (800 parasites/µL) (A) and correlation between C_t_ value and parasitemia (B). (A) Plot of the mean cycle threshold (C_t_) values against *P. vivax* DNA amount extracted from blood samples with a parasitemia of 800 parasites/µL using 2-fold dilutions (2^0^–2^13^ dilutions). The plot of C_t_ values and DNA amount fits a linear function (*R* ^2^ = 0.9703). (B) Log values of parasitemia (parasites/µL) against C_t_ values were plotted for 54 blood samples infected with *P. vivax*.

First, the real-time PCR reaction products and program were optimized, and then the method was validated. The specificity of the real-time PCR assay was confirmed using samples infected with *P. vivax*, *P. falciparum*, *P. malariae*, *P. ovale* and samples from normal healthy individuals. No amplification was observed in *P. falciparum*, *P. malariae*, *P. ovale* controls, or 10 negative control samples (data not shown). The samples with a parasitemia of 800 parasites/µL showed reproducible linearity of >1,024-fold determined with C_t_ values. As shown in Figure 1, a significant correlation coefficient was found between the mean C_t_ values and parasitemia in a diluted sample (*R* ^2^ = 0.9703) as well as in samples collected from 54 vivax malaria patients. This LOD of real-time PCR was similar to that of nested PCR (0.78 parasites/µL) in the sample with 800 parasites/µL (Table 2).

**Table 2:** Performance of four PCR-based assays—real-time PCR, nested PCR, LAMP, and multiplex PCR—and microscopic examination for vivax malaria diagnosis.

	Real-time PCR	Nested PCR	LAMP	Multiplex PCR	Microscopic examination
Positive (%)	273 (85.6)	274 (85.9)	273 (85.6)	273 (85.6)	54 (28.1)
Negative (%)	46 (14.4)	45 (14.1)	46 (14.4)	46 (14.4)	138 (71.9)
Total	319	319	319	319	192
LOD (parasites/µL)	0.78	0.78	0.78	1.56	

LAMP: loop-mediated isothermal amplification; PCR: polymerase chain reaction; LOD: limit of detection.

Considering the suggestion by Gama et al. [28] that clinical samples of *P. vivax* infection with similar parasitemia have various copy numbers of rRNA genes owing to different proportions of schizonts, which leads to variation in real-time PCR parasite quantification, we normalized the parasite number according to the schizont proportion. As shown in Figure 1, the standard curve generated from *P. vivax* genomic DNA showed a significant correlation between mean Ct value and parasite density, similar to that of another standard curve drawn with a synthetic amplicon [24]. Except for the multiplex PCR method (1.56 parasites/µL), the remaining 3 PCR-based assays—real-time PCR, nested PCR, and LAMP—had the same LOD (0.78 parasites/µL). This result suggests that the developed real-time PCR method is as sensitive as the other PCR-based methods. In addition, LOD is approximately 10 times more sensitive than microscopic examination performed by experienced microscopists (10–50 parasites/µL) [29].

All 319 samples suspected to be vivax malaria-positive were tested using the 4 PCR-based assays. A total of 274, 273, 273, and 273 samples were identified as positive, and 45, 46, 46, and 46 samples were identified as negative using nested PCR, real-time PCR, LAMP, and multiplex PCR, respectively (Table 2). Sensitivity, specificity, and agreement of each test method were calculated using nested PCR results as a reference standard (Table 3). As demonstrated by the 95% confidence intervals, real-time PCR, LAMP, and multiplex PCR showed high sensitivity and specificity. Real-time PCR and LAMP (both with a sensitivity of 99.6% and specificity of 100%) were slightly more sensitive and specific than multiplex PCR (sensitivity of 99.3% and specificity of 99.1%). However, the differences in sensitivity and specificity were not significant among these 3 PCR-based assays. Furthermore, these assays showed high agreement (99.1%–99.9%) with nested PCR results.

**Table 3:** Sensitivity, specificity, and agreement of real-time PCR, LAMP, and multiplex PCR using nested PCR results as the gold standard.

	Real-time PCR	LAMP	Multiplex PCR
Sensitivity^a^ (%)	99.6 (97.7–100)	99.6 (97.7–100)	99.3 (97.1–99.9)
Specificity^a^ (%)	100 (90.2–100)	100 (90.2–100)	97.8 (86.8–99.9)
Agreement^a,b^ (%)	99.9 (98.0–100)	99.9 (98.0–100)	99.1 (97.0–99.8)

a: 95% confidence intervals are shown in parentheses.

b: calculated by DAG_Stat and VassarStats (http://vassarstats.net/).

LAMP: loop-mediated isothermal amplification; PCR: polymerase chain reaction.

Nested PCR has been reported as a specific and sensitive diagnostic method to detect vivax malaria; however, the assay is inadequate for use in a clinical setting because the turnaround time is incompatible with the urgency required to initiate treatment. By contrast, the results of real-time PCR and LAMP can be obtained in only 2 hours. Furthermore, these two methods can be used to quantify parasite load with less labor and result in lower risk of contamination after DNA amplification. In terms of cost, microscopy is the most appropriate method for vivax malaria diagnosis. However, as shown in Table S1, it is less sensitive in samples with low parasitemia. The simplified step in both real-time PCR and LAMP (i.e., no gel electrophoresis and only one PCR analysis are required) makes these techniques cheaper compared with nested PCR. Indeed, LAMP can be performed even without a thermocycler. These advantages suggest that real-time PCR or LAMP could be used for vivax parasite detection in medical clinics.

The WHO recommends microscopic examination as the gold standard for the diagnosis of vivax malaria, although an immunochromatography-based rapid diagnostic test (RDT) has been suggested as an alternative [1]. However, RDTs often misdiagnose malaria in samples with low parasitemia (<100 parasites/µL), which could hamper the accurate diagnosis of vivax malaria in the ROK, where patients usually have low parasitemia [30]. Therefore, PCR-based assays can be valuable tools when malaria cases with low parasitemia need to be identified—for example, when screening blood donors in endemic areas or even monitoring malaria treatment.

In conclusion, we established a real-time PCR method that can detect *P. vivax* in field samples. Compared with nested PCR, LAMP, and multiplex PCR, our real-time PCR assay is a sensitive, specific, rapid, and quantitative method. In addition, our results showed that PCR-based assays, including real-time PCR, could be useful in the diagnosis of vivax malaria in the ROK, where most vivax malaria patients have low parasitemia.

## Supporting Information

Table S1 **Performance of four PCR-based assays compared with results of microscopic examination for vivax malaria diagnosis in samples with malaria symptoms.**
(DOC)
