# Supplementary material for: Development and Efficacy of Real-Time PCR in the Diagnosis of Vivax Malaria Using Field Samples in the Republic of Korea
Source: PLoS One. 2014 Aug 22;9(8):e105871. doi: 10.1371/journal.pone.0105871 (PMC4141806; doi:10.1371/journal.pone.0105871)
Supplement: Table S1 — Performance of four PCR-based assays compared with results of microscopic examination for vivax malaria diagnosis in samples with malaria symptoms. (DOC) [file pone.0105871.s001.doc]

|  |  |  |  |  | |  |  |  |  |  |  | |
| --- | --- | --- | --- | --- | --- | --- | --- | --- | --- | --- | --- | --- |
|  | Nested PCR | |  | Real-time PCR | | |  | LAMP | |  | Multiplex PCR | |
| Microscopic examination | Positive  (%) | Negative  (%) |  | Positive  (%) | Negative  (%) | |  | Positive  (%) | Negative  (%) |  | Positive  (%) | Negative  (%) |
| Positive  (%) | 54  (28.1) | 0 |  | 54  (28.1) | 0 | |  | 54  (28.1) | 0 |  | 54  (28.1) | 0 |
| Negative  (%) | 113  (58.9) | 25  (13.0) |  | 112  (58.3) | 26  (13.6) | |  | 113  (58.9) | 25  (13.0) |  | 112  (58.3) | 26  (13.6) |
|  |  |  |  |  |  | |  |  |  |  |  |  |

Table S1. Performance of four PCR-based assays compared with results of microscopic examination for vivax malaria diagnosis in samples with malaria symptoms.
